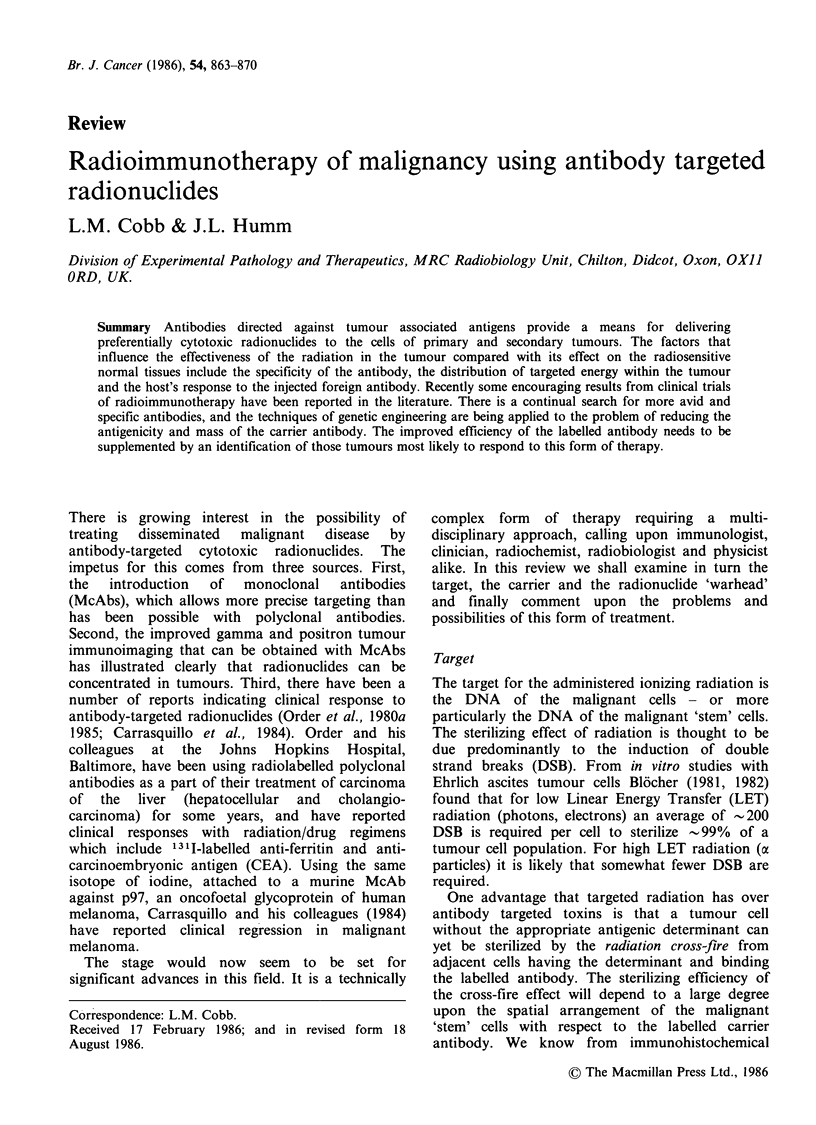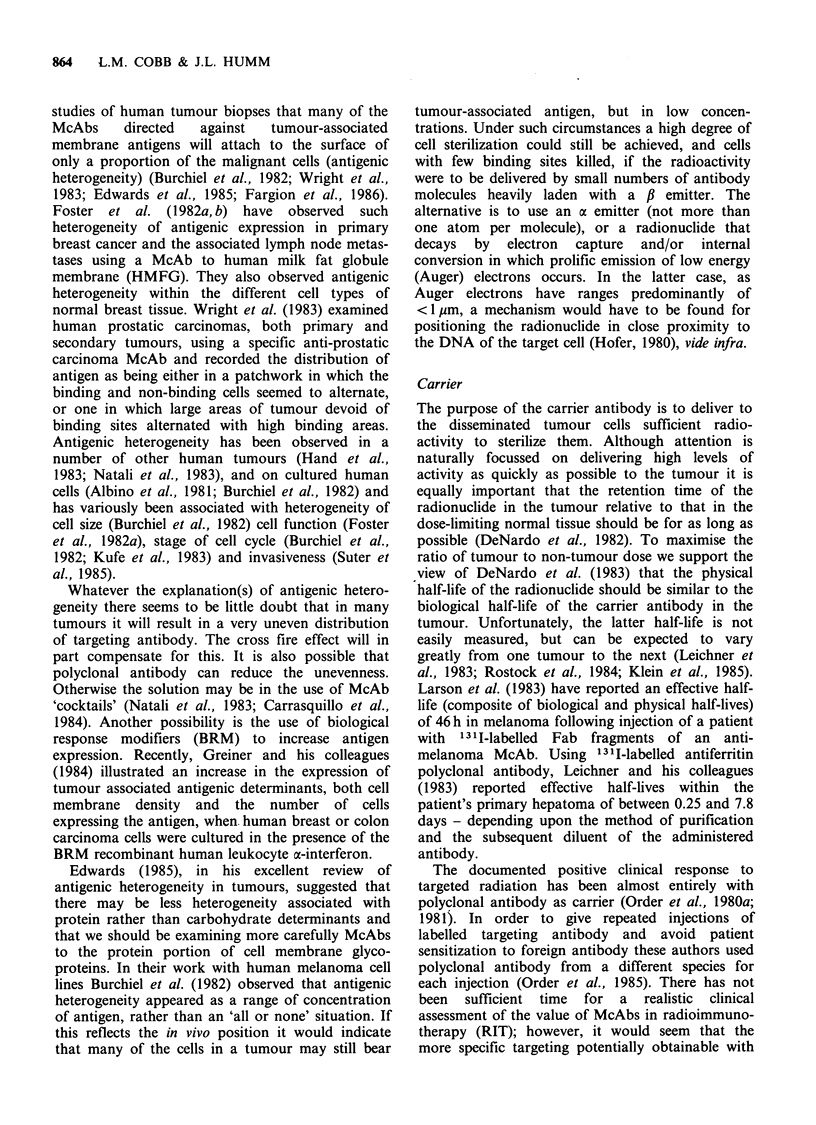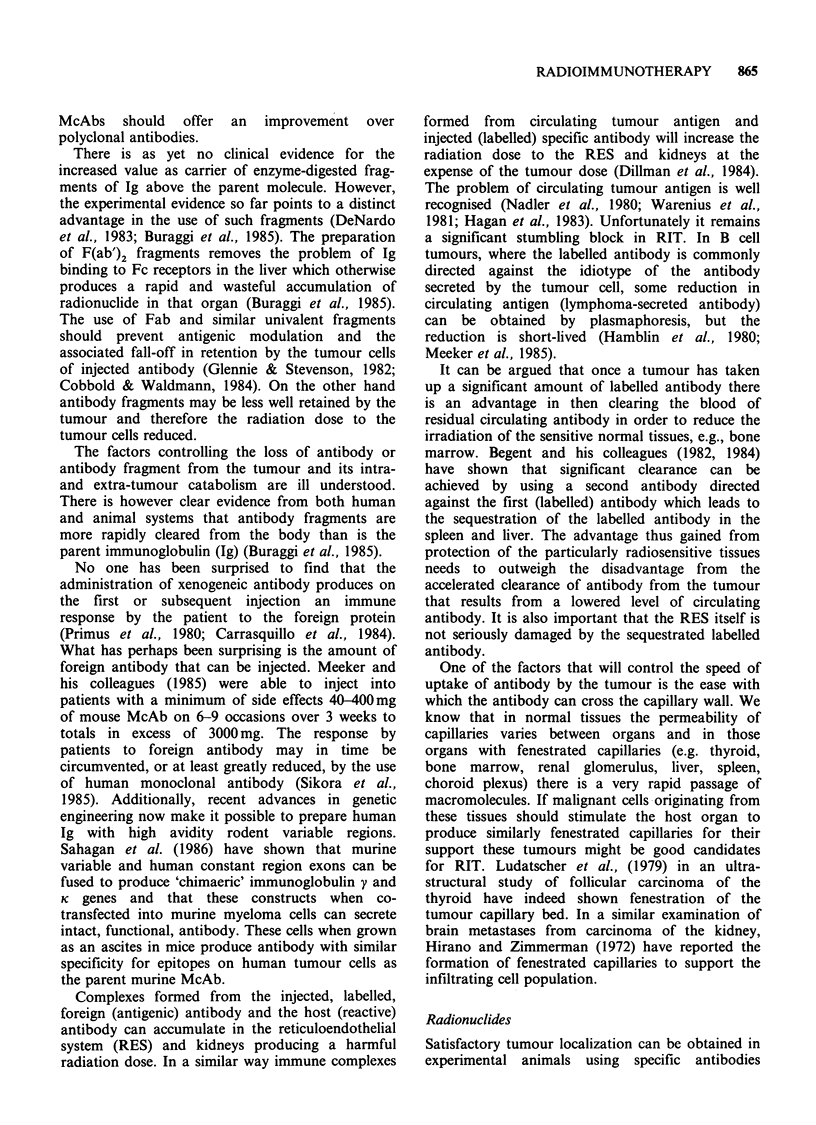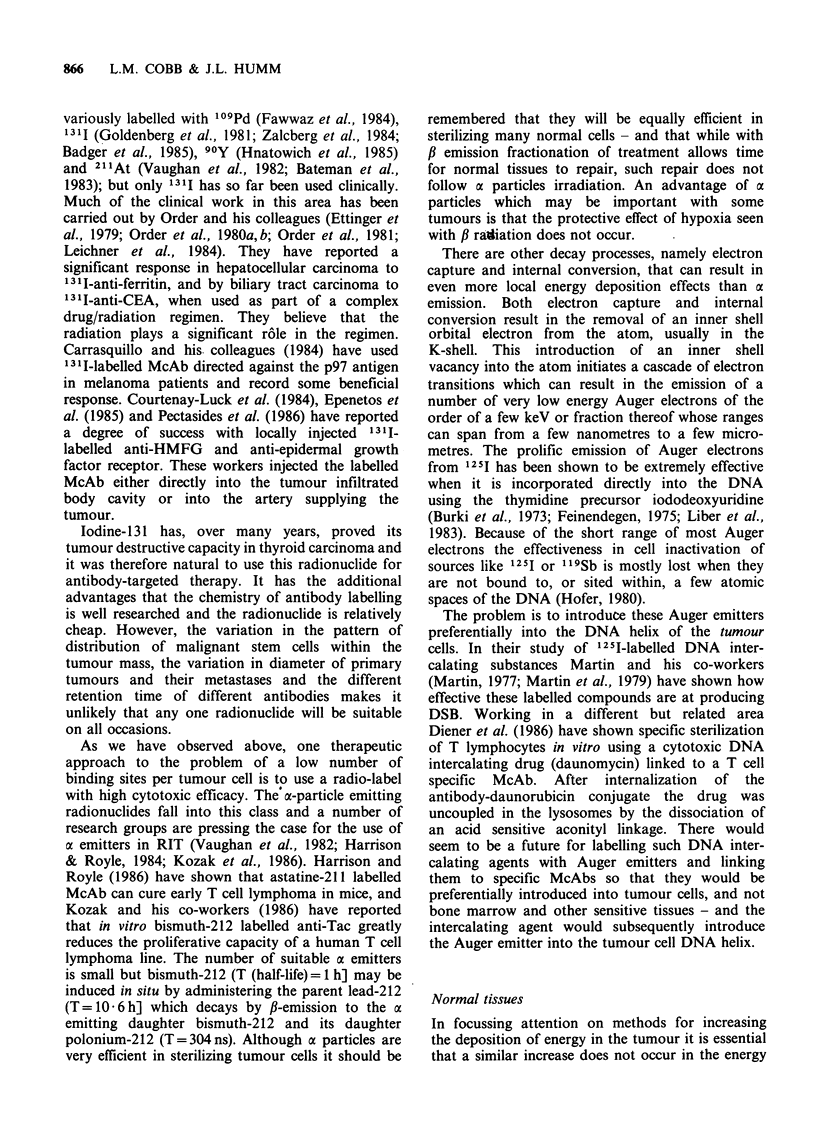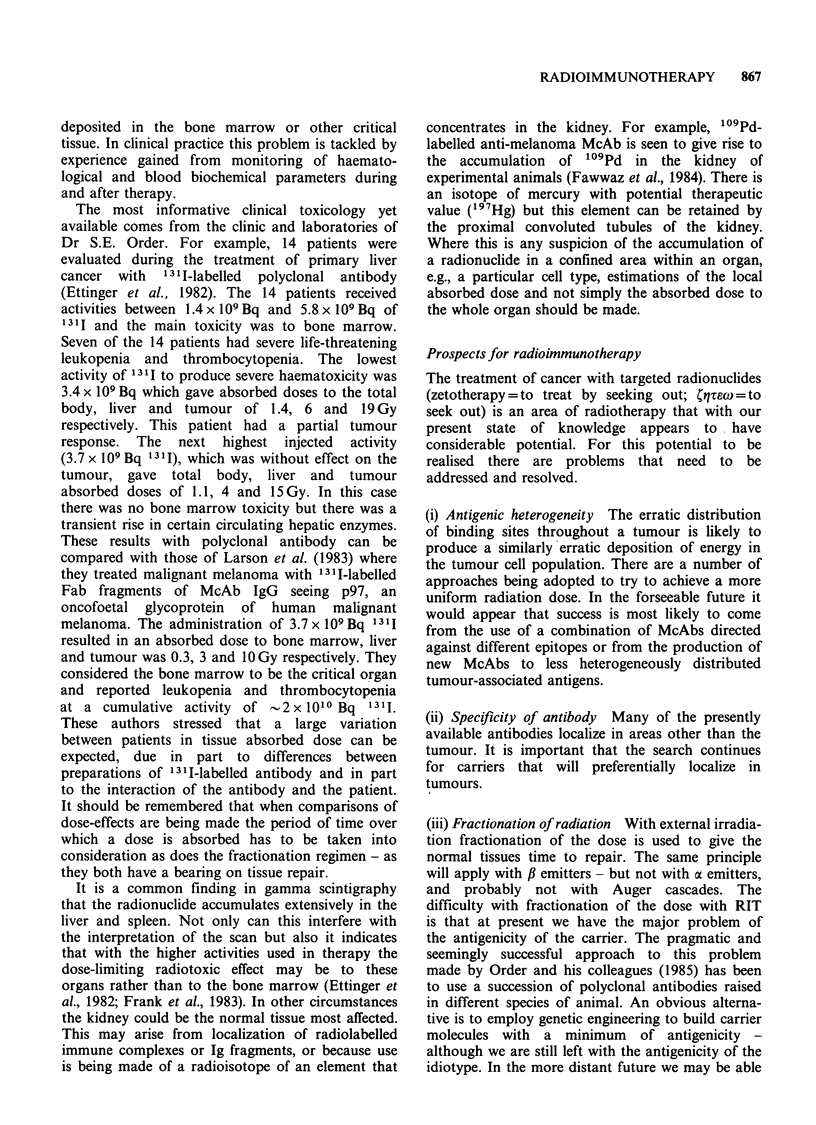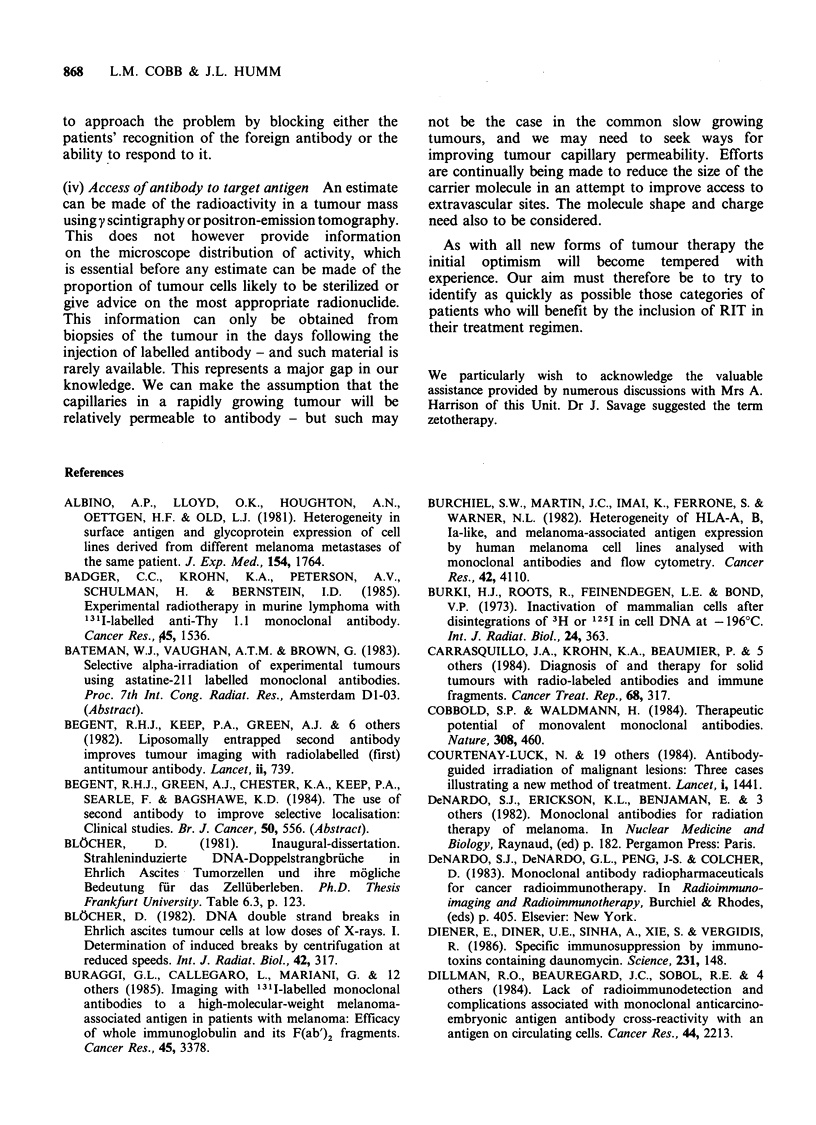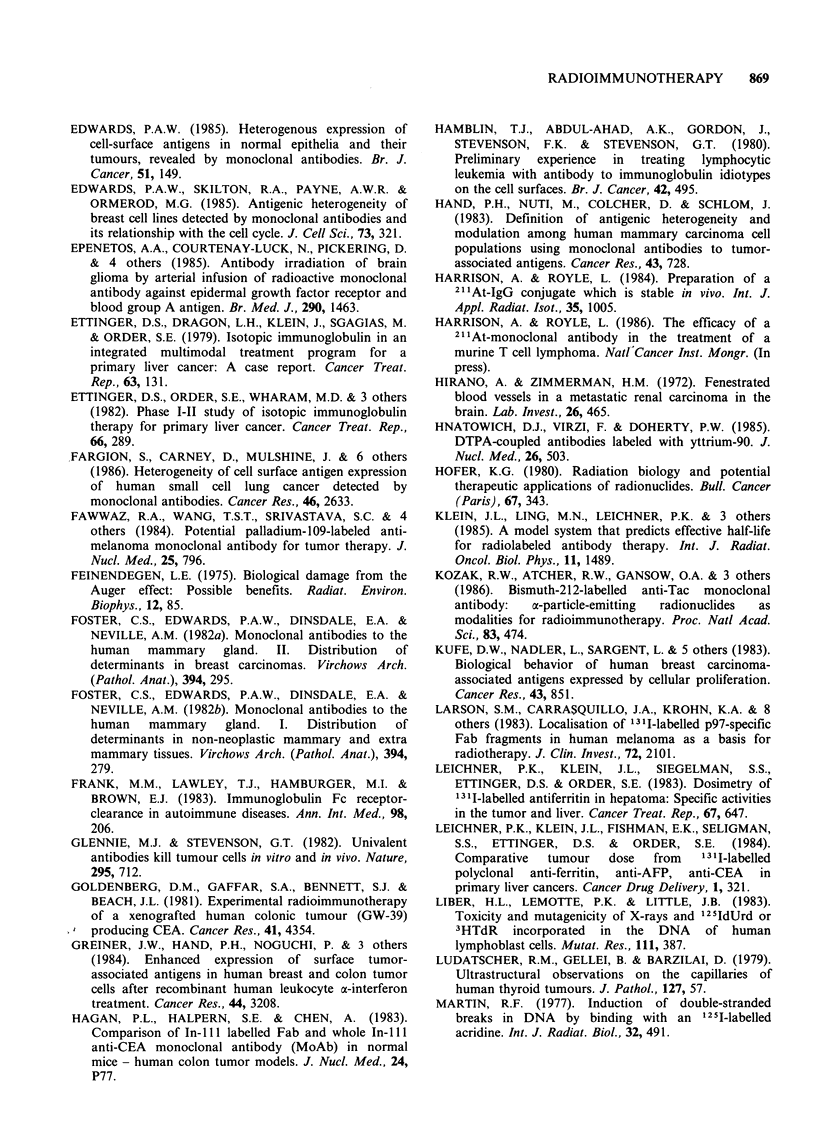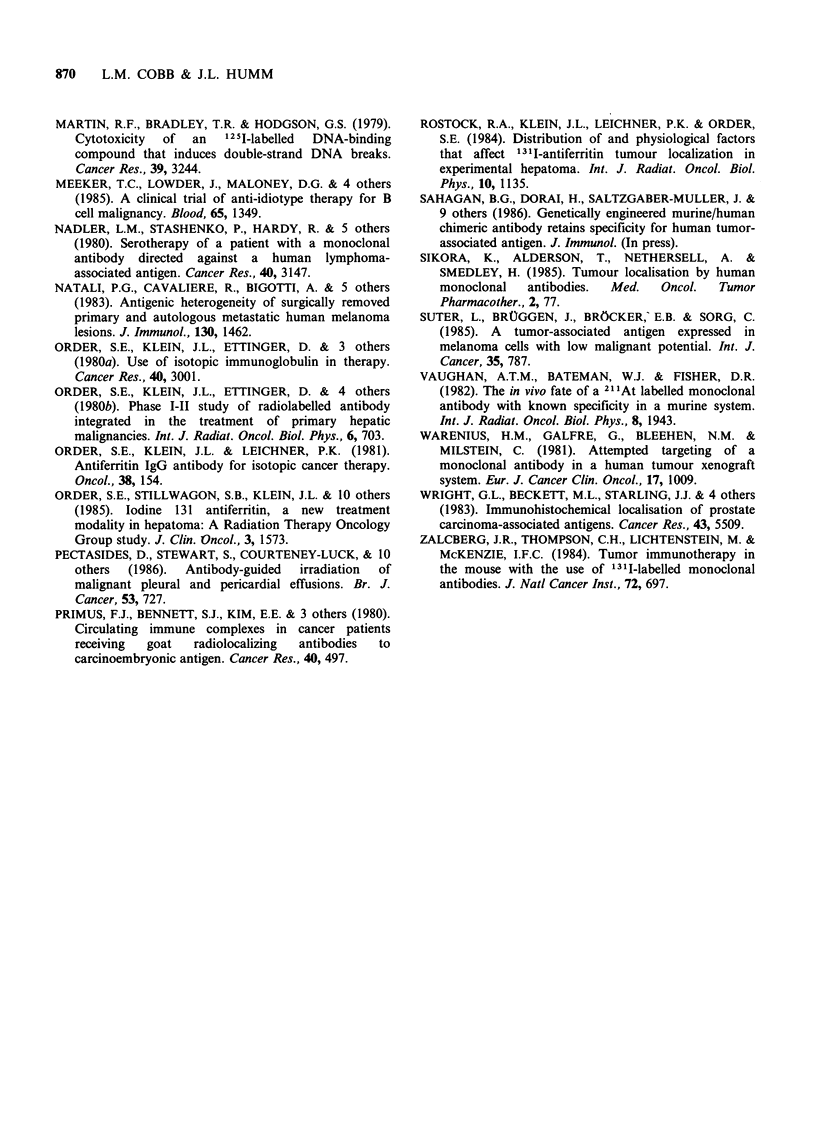# Radioimmunotherapy of malignancy using antibody targeted radionuclides.

**DOI:** 10.1038/bjc.1986.254

**Published:** 1986-12

**Authors:** L. M. Cobb, J. L. Humm

## Abstract

Antibodies directed against tumour associated antigens provide a means for delivering preferentially cytotoxic radionuclides to the cells of primary and secondary tumours. The factors that influence the effectiveness of the radiation in the tumour compared with its effect on the radiosensitive normal tissues include the specificity of the antibody, the distribution of targeted energy within the tumour and the host's response to the injected foreign antibody. Recently some encouraging results from clinical trials of radioimmunotherapy have been reported in the literature. There is a continual search for more avid and specific antibodies, and the techniques of genetic engineering are being applied to the problem of reducing the antigenicity and mass of the carrier antibody. The improved efficiency of the labelled antibody needs to be supplemented by an identification of those tumours most likely to respond to this form of therapy.


*Br. J. Cancer* (1986), **54**, 863–870

### Review

### Radioimmunotherapy of malignancy using antibody targeted radionuclides

L.M. Cobb & J.L. Humm

*Division of Experimental Pathology and Therapeutics, MRC Radiobiology Unit, Chilton, Didcot, Oxon, OX11 0RD, UK.*

**Summary** Antibodies directed against tumour associated antigens provide a means for delivering preferentially cytotoxic radionuclides to the cells of primary and secondary tumours. The factors that influence the effectiveness of the radiation in the tumour compared with its effect on the radiosensitive normal tissues include the specificity of the antibody, the distribution of targeted energy within the tumour and the host's response to the injected foreign antibody. Recently some encouraging results from clinical trials of radioimmunotherapy have been reported in the literature. There is a continual search for more avid and specific antibodies, and the techniques of genetic engineering are being applied to the problem of reducing the antigenicity and mass of the carrier antibody. The improved efficiency of the labelled antibody needs to be supplemented by an identification of those tumours most likely to respond to this form of therapy.

There is growing interest in the possibility of treating disseminated malignant disease by antibody-targeted cytotoxic radionuclides. The impetus for this comes from three sources. First, the introduction of monoclonal antibodies (McAbs), which allows more precise targeting than has been possible with polyclonal antibodies. Second, the improved gamma and positron tumour immunoimaging that can be obtained with McAbs has illustrated clearly that radionuclides can be concentrated in tumours. Third, there have been a number of reports indicating clinical response to antibody-targeted radionuclides (Order *et al.*, 1980*a* 1985; Carrasquillo *et al.*, 1984). Order and his colleagues at the Johns Hopkins Hospital, Baltimore, have been using radiolabelled polyclonal antibodies as a part of their treatment of carcinoma of the liver (hepatocellular and cholangiocarcinoma) for some years, and have reported clinical responses with radiation/drug regimens which include $^{131}$I-labelled anti-ferritin and anti-carcinoembryonic antigen (CEA). Using the same isotope of iodine, attached to a murine McAb against p97, an oncofoetal glycoprotein of human melanoma, Carrasquillo and his colleagues (1984) have reported clinical regression in malignant melanoma.

The stage would now seem to be set for significant advances in this field. It is a technically complex form of therapy requiring a multi-disciplinary approach, calling upon immunologist, clinician, radiochemist, radiobiologist and physicist alike. In this review we shall examine in turn the target, the carrier and the radionuclide 'warhead' and finally comment upon the problems and possibilities of this form of treatment.

### *Target*

The target for the administered ionizing radiation is the DNA of the malignant cells – or more particularly the DNA of the malignant 'stem' cells. The sterilizing effect of radiation is thought to be due predominantly to the induction of double strand breaks (DSB). From *in vitro* studies with Ehrlich ascites tumour cells Blöcher (1981, 1982) found that for low Linear Energy Transfer (LET) radiation (photons, electrons) an average of $\sim$200 DSB is required per cell to sterilize $\sim$99% of a tumour cell population. For high LET radiation ($\alpha$ particles) it is likely that somewhat fewer DSB are required.

One advantage that targeted radiation has over antibody targeted toxins is that a tumour cell without the appropriate antigenic determinant can yet be sterilized by the *radiation cross-fire* from adjacent cells having the determinant and binding the labelled antibody. The sterilizing efficiency of the cross-fire effect will depend to a large degree upon the spatial arrangement of the malignant 'stem' cells with respect to the labelled carrier antibody. We know from immunohistochemical

Correspondence: L.M. Cobb.
Received 17 February 1986; and in revised form 18 August 1986.

© The Macmillan Press Ltd., 1986

studies of human tumour biopses that many of the McAbs directed against tumour-associated membrane antigens will attach to the surface of only a proportion of the malignant cells (antigenic heterogeneity) (Burchiel *et al.*, 1982; Wright *et al.*, 1983; Edwards *et al.*, 1985; Fargion *et al.*, 1986). Foster *et al.* (1982*a,b*) have observed such heterogeneity of antigenic expression in primary breast cancer and the associated lymph node metastases using a McAb to human milk fat globule membrane (HMFG). They also observed antigenic heterogeneity within the different cell types of normal breast tissue. Wright *et al.* (1983) examined human prostatic carcinomas, both primary and secondary tumours, using a specific anti-prostatic carcinoma McAb and recorded the distribution of antigen as being either in a patchwork in which the binding and non-binding cells seemed to alternate, or one in which large areas of tumour devoid of binding sites alternated with high binding areas. Antigenic heterogeneity has been observed in a number of other human tumours (Hand *et al.*, 1983; Natali *et al.*, 1983), and on cultured human cells (Albino *et al.*, 1981; Burchiel *et al.*, 1982) and has variously been associated with heterogeneity of cell size (Burchiel *et al.*, 1982) cell function (Foster *et al.*, 1982*a*), stage of cell cycle (Burchiel *et al.*, 1982; Kufe *et al.*, 1983) and invasiveness (Suter *et al.*, 1985).

Whatever the explanation(s) of antigenic heterogeneity there seems to be little doubt that in many tumours it will result in a very uneven distribution of targeting antibody. The cross fire effect will in part compensate for this. It is also possible that polyclonal antibody can reduce the unevenness. Otherwise the solution may be in the use of McAb 'cocktails' (Natali *et al.*, 1983; Carrasquillo *et al.*, 1984). Another possibility is the use of biological response modifiers (BRM) to increase antigen expression. Recently, Greiner and his colleagues (1984) illustrated an increase in the expression of tumour associated antigenic determinants, both cell membrane density and the number of cells expressing the antigen, when human breast or colon carcinoma cells were cultured in the presence of the BRM recombinant human leukocyte α-interferon.

Edwards (1985), in his excellent review of antigenic heterogeneity in tumours, suggested that there may be less heterogeneity associated with protein rather than carbohydrate determinants and that we should be examining more carefully McAbs to the protein portion of cell membrane glycoproteins. In their work with human melanoma cell lines Burchiel *et al.* (1982) observed that antigenic heterogeneity appeared as a range of concentration of antigen, rather than an 'all or none' situation. If this reflects the *in vivo* position it would indicate that many of the cells in a tumour may still bear tumour-associated antigen, but in low concentrations. Under such circumstances a high degree of cell sterilization could still be achieved, and cells with few binding sites killed, if the radioactivity were to be delivered by small numbers of antibody molecules heavily laden with a $\beta$ emitter. The alternative is to use an $\alpha$ emitter (not more than one atom per molecule), or a radionuclide that decays by electron capture and/or internal conversion in which prolific emission of low energy (Auger) electrons occurs. In the latter case, as Auger electrons have ranges predominantly of $<1\,\mu$m, a mechanism would have to be found for positioning the radionuclide in close proximity to the DNA of the target cell (Hofer, 1980), *vide infra*.

*Carrier*

The purpose of the carrier antibody is to deliver to the disseminated tumour cells sufficient radioactivity to sterilize them. Although attention is naturally focussed on delivering high levels of activity as quickly as possible to the tumour it is equally important that the retention time of the radionuclide in the tumour relative to that in the dose-limiting normal tissue should be for as long as possible (DeNardo *et al.*, 1982). To maximise the ratio of tumour to non-tumour dose we support the view of DeNardo *et al.* (1983) that the physical half-life of the radionuclide should be similar to the biological half-life of the carrier antibody in the tumour. Unfortunately, the latter half-life is not easily measured, but can be expected to vary greatly from one tumour to the next (Leichner *et al.*, 1983; Rostock *et al.*, 1984; Klein *et al.*, 1985). Larson *et al.* (1983) have reported an effective half-life (composite of biological and physical half-lives) of 46 h in melanoma following injection of a patient with $^{131}$I-labelled Fab fragments of an anti-melanoma McAb. Using $^{131}$I-labelled antiferritin polyclonal antibody, Leichner and his colleagues (1983) reported effective half-lives within the patient's primary hepatoma of between 0.25 and 7.8 days – depending upon the method of purification and the subsequent diluent of the administered antibody.

The documented positive clinical response to targeted radiation has been almost entirely with polyclonal antibody as carrier (Order *et al.*, 1980*a*; 1981). In order to give repeated injections of labelled targeting antibody and avoid patient sensitization to foreign antibody these authors used polyclonal antibody from a different species for each injection (Order *et al.*, 1985). There has not been sufficient time for a realistic clinical assessment of the value of McAbs in radioimmunotherapy (RIT); however, it would seem that the more specific targeting potentially obtainable with

McAbs should offer an improvement over polyclonal antibodies.

There is as yet no clinical evidence for the increased value as carrier of enzyme-digested fragments of Ig above the parent molecule. However, the experimental evidence so far points to a distinct advantage in the use of such fragments (DeNardo *et al.*, 1983; Buraggi *et al.*, 1985). The preparation of $F(ab')_2$ fragments removes the problem of Ig binding to Fc receptors in the liver which otherwise produces a rapid and wasteful accumulation of radionuclide in that organ (Buraggi *et al.*, 1985). The use of Fab and similar univalent fragments should prevent antigenic modulation and the associated fall-off in retention by the tumour cells of injected antibody (Glennie & Stevenson, 1982; Cobbold & Waldmann, 1984). On the other hand antibody fragments may be less well retained by the tumour and therefore the radiation dose to the tumour cells reduced.

The factors controlling the loss of antibody or antibody fragment from the tumour and its intra- and extra-tumour catabolism are ill understood. There is however clear evidence from both human and animal systems that antibody fragments are more rapidly cleared from the body than is the parent immunoglobulin (Ig) (Buraggi *et al.*, 1985).

No one has been surprised to find that the administration of xenogeneic antibody produces on the first or subsequent injection an immune response by the patient to the foreign protein (Primus *et al.*, 1980; Carrasquillo *et al.*, 1984). What has perhaps been surprising is the amount of foreign antibody that can be injected. Meeker and his colleagues (1985) were able to inject into patients with a minimum of side effects 40–400 mg of mouse McAb on 6–9 occasions over 3 weeks to totals in excess of 3000 mg. The response by patients to foreign antibody may in time be circumvented, or at least greatly reduced, by the use of human monoclonal antibody (Sikora *et al.*, 1985). Additionally, recent advances in genetic engineering now make it possible to prepare human Ig with high avidity rodent variable regions. Sahagan *et al.* (1986) have shown that murine variable and human constant region exons can be fused to produce 'chimaeric' immunoglobulin $\gamma$ and $\kappa$ genes and that these constructs when co-transfected into murine myeloma cells can secrete intact, functional, antibody. These cells when grown as an ascites in mice produce antibody with similar specificity for epitopes on human tumour cells as the parent murine McAb.

Complexes formed from the injected, labelled, foreign (antigenic) antibody and the host (reactive) antibody can accumulate in the reticuloendothelial system (RES) and kidneys producing a harmful radiation dose. In a similar way immune complexes formed from circulating tumour antigen and injected (labelled) specific antibody will increase the radiation dose to the RES and kidneys at the expense of the tumour dose (Dillman *et al.*, 1984). The problem of circulating tumour antigen is well recognised (Nadler *et al.*, 1980; Warenius *et al.*, 1981; Hagan *et al.*, 1983). Unfortunately it remains a significant stumbling block in RIT. In B cell tumours, where the labelled antibody is commonly directed against the idiotype of the antibody secreted by the tumour cell, some reduction in circulating antigen (lymphoma-secreted antibody) can be obtained by plasmaphoresis, but the reduction is short-lived (Hamblin *et al.*, 1980; Meeker *et al.*, 1985).

It can be argued that once a tumour has taken up a significant amount of labelled antibody there is an advantage in then clearing the blood of residual circulating antibody in order to reduce the irradiation of the sensitive normal tissues, e.g., bone marrow. Begent and his colleagues (1982, 1984) have shown that significant clearance can be achieved by using a second antibody directed against the first (labelled) antibody which leads to the sequestration of the labelled antibody in the spleen and liver. The advantage thus gained from protection of the particularly radiosensitive tissues needs to outweigh the disadvantage from the accelerated clearance of antibody from the tumour that results from a lowered level of circulating antibody. It is also important that the RES itself is not seriously damaged by the sequestrated labelled antibody.

One of the factors that will control the speed of uptake of antibody by the tumour is the ease with which the antibody can cross the capillary wall. We know that in normal tissues the permeability of capillaries varies between organs and in those organs with fenestrated capillaries (e.g. thyroid, bone marrow, renal glomerulus, liver, spleen, choroid plexus) there is a very rapid passage of macromolecules. If malignant cells originating from these tissues should stimulate the host organ to produce similarly fenestrated capillaries for their support these tumours might be good candidates for RIT. Ludatscher *et al.*, (1979) in an ultra-structural study of follicular carcinoma of the thyroid have indeed shown fenestration of the tumour capillary bed. In a similar examination of brain metastases from carcinoma of the kidney, Hirano and Zimmerman (1972) have reported the formation of fenestrated capillaries to support the infiltrating cell population.

### *Radionuclides*

Satisfactory tumour localization can be obtained in experimental animals using specific antibodies variously labelled with $^{109}$Pd (Fawwaz *et al.*, 1984), $^{131}$I (Goldenberg *et al.*, 1981; Zalcberg *et al.*, 1984; Badger *et al.*, 1985), $^{90}$Y (Hnatowich *et al.*, 1985) and $^{211}$At (Vaughan *et al.*, 1982; Bateman *et al.*, 1983); but only $^{131}$I has so far been used clinically. Much of the clinical work in this area has been carried out by Order and his colleagues (Ettinger *et al.*, 1979; Order *et al.*, 1980*a,b*; Order *et al.*, 1981; Leichner *et al.*, 1984). They have reported a significant response in hepatocellular carcinoma to $^{131}$I-anti-ferritin, and by biliary tract carcinoma to $^{131}$I-anti-CEA, when used as part of a complex drug/radiation regimen. They believe that the radiation plays a significant rôle in the regimen. Carrasquillo and his colleagues (1984) have used $^{131}$I-labelled McAb directed against the p97 antigen in melanoma patients and record some beneficial response. Courtenay-Luck *et al.* (1984), Epenetos *et al.* (1985) and Pectasides *et al.* (1986) have reported a degree of success with locally injected $^{131}$I-labelled anti-HMFG and anti-epidermal growth factor receptor. These workers injected the labelled McAb either directly into the tumour infiltrated body cavity or into the artery supplying the tumour.

Iodine-131 has, over many years, proved its tumour destructive capacity in thyroid carcinoma and it was therefore natural to use this radionuclide for antibody-targeted therapy. It has the additional advantages that the chemistry of antibody labelling is well researched and the radionuclide is relatively cheap. However, the variation in the pattern of distribution of malignant stem cells within the tumour mass, the variation in diameter of primary tumours and their metastases and the different retention time of different antibodies makes it unlikely that any one radionuclide will be suitable on all occasions.

As we have observed above, one therapeutic approach to the problem of a low number of binding sites per tumour cell is to use a radio-label with high cytotoxic efficacy. The $\alpha$-particle emitting radionuclides fall into this class and a number of research groups are pressing the case for the use of $\alpha$ emitters in RIT (Vaughan *et al.*, 1982; Harrison & Royle, 1984; Kozak *et al.*, 1986). Harrison and Royle (1986) have shown that astatine-211 labelled McAb can cure early T cell lymphoma in mice, and Kozak and his co-workers (1986) have reported that *in vitro* bismuth-212 labelled anti-Tac greatly reduces the proliferative capacity of a human T cell lymphoma line. The number of suitable $\alpha$ emitters is small but bismuth-212 (T (half-life) = 1 h] may be induced *in situ* by administering the parent lead-212 (T = 10·6 h] which decays by $\beta$-emission to the $\alpha$ emitting daughter bismuth-212 and its daughter polonium-212 (T = 304 ns). Although $\alpha$ particles are very efficient in sterilizing tumour cells it should be remembered that they will be equally efficient in sterilizing many normal cells – and that while with $\beta$ emission fractionation of treatment allows time for normal tissues to repair, such repair does not follow $\alpha$ particles irradiation. An advantage of $\alpha$ particles which may be important with some tumours is that the protective effect of hypoxia seen with $\beta$ radiation does not occur.

There are other decay processes, namely electron capture and internal conversion, that can result in even more local energy deposition effects than $\alpha$ emission. Both electron capture and internal conversion result in the removal of an inner shell orbital electron from the atom, usually in the K-shell. This introduction of an inner shell vacancy into the atom initiates a cascade of electron transitions which can result in the emission of a number of very low energy Auger electrons of the order of a few keV or fraction thereof whose ranges can span from a few nanometres to a few micrometres. The prolific emission of Auger electrons from $^{125}$I has been shown to be extremely effective when it is incorporated directly into the DNA using the thymidine precursor iododeoxyuridine (Burki *et al.*, 1973; Feinendegen, 1975; Liber *et al.*, 1983). Because of the short range of most Auger electrons the effectiveness in cell inactivation of sources like $^{125}$I or $^{119}$Sb is mostly lost when they are not bound to, or sited within, a few atomic spaces of the DNA (Hofer, 1980).

The problem is to introduce these Auger emitters preferentially into the DNA helix of the *tumour* cells. In their study of $^{125}$I-labelled DNA intercalating substances Martin and his co-workers (Martin, 1977; Martin *et al.*, 1979) have shown how effective these labelled compounds are at producing DSB. Working in a different but related area Diener *et al.* (1986) have shown specific sterilization of T lymphocytes *in vitro* using a cytotoxic DNA intercalating drug (daunomycin) linked to a T cell specific McAb. After internalization of the antibody-daunorubicin conjugate the drug was uncoupled in the lysosomes by the dissociation of an acid sensitive aconityl linkage. There would seem to be a future for labelling such DNA intercalating agents with Auger emitters and linking them to specific McAbs so that they would be preferentially introduced into tumour cells, and not bone marrow and other sensitive tissues – and the intercalating agent would subsequently introduce the Auger emitter into the tumour cell DNA helix.

### *Normal tissues*

In focussing attention on methods for increasing the deposition of energy in the tumour it is essential that a similar increase does not occur in the energy deposited in the bone marrow or other critical tissue. In clinical practice this problem is tackled by experience gained from monitoring of haematological and blood biochemical parameters during and after therapy.

The most informative clinical toxicology yet available comes from the clinic and laboratories of Dr S.E. Order. For example, 14 patients were evaluated during the treatment of primary liver cancer with $^{131}$I-labelled polyclonal antibody (Ettinger *et al.,* 1982). The 14 patients received activities between $1.4 \times 10^9$ Bq and $5.8 \times 10^9$ Bq of $^{131}$I and the main toxicity was to bone marrow. Seven of the 14 patients had severe life-threatening leukopenia and thrombocytopenia. The lowest activity of $^{131}$I to produce severe haematoxicity was $3.4 \times 10^9$ Bq which gave absorbed doses to the total body, liver and tumour of 1.4, 6 and 19 Gy respectively. This patient had a partial tumour response. The next highest injected activity ($3.7 \times 10^9$ Bq $^{131}$I), which was without effect on the tumour, gave total body, liver and tumour absorbed doses of 1.1, 4 and 15 Gy. In this case there was no bone marrow toxicity but there was a transient rise in certain circulating hepatic enzymes. These results with polyclonal antibody can be compared with those of Larson *et al.* (1983) where they treated malignant melanoma with $^{131}$I-labelled Fab fragments of McAb IgG seeing p97, an oncofoetal glycoprotein of human malignant melanoma. The administration of $3.7 \times 10^9$ Bq $^{131}$I resulted in an absorbed dose to bone marrow, liver and tumour was 0.3, 3 and 10 Gy respectively. They considered the bone marrow to be the critical organ and reported leukopenia and thrombocytopenia at a cumulative activity of $\sim 2 \times 10^{10}$ Bq $^{131}$I. These authors stressed that a large variation between patients in tissue absorbed dose can be expected, due in part to differences between preparations of $^{131}$I-labelled antibody and in part to the interaction of the antibody and the patient. It should be remembered that when comparisons of dose-effects are being made the period of time over which a dose is absorbed has to be taken into consideration as does the fractionation regimen – as they both have a bearing on tissue repair.

It is a common finding in gamma scintigraphy that the radionuclide accumulates extensively in the liver and spleen. Not only can this interfere with the interpretation of the scan but also it indicates that with the higher activities used in therapy the dose-limiting radiotoxic effect may be to these organs rather than to the bone marrow (Ettinger *et al.*, 1982; Frank *et al.*, 1983). In other circumstances the kidney could be the normal tissue most affected. This may arise from localization of radiolabelled immune complexes or Ig fragments, or because use is being made of a radioisotope of an element that concentrates in the kidney. For example, $^{109}$Pd-labelled anti-melanoma McAb is seen to give rise to the accumulation of $^{109}$Pd in the kidney of experimental animals (Fawwaz *et al.*, 1984). There is an isotope of mercury with potential therapeutic value ($^{197}$Hg) but this element can be retained by the proximal convoluted tubules of the kidney. Where this is any suspicion of the accumulation of a radionuclide in a confined area within an organ, e.g., a particular cell type, estimations of the local absorbed dose and not simply the absorbed dose to the whole organ should be made.

### *Prospects for radioimmunotherapy*

The treatment of cancer with targeted radionuclides (zetotherapy = to treat by seeking out; ζητεω = to seek out) is an area of radiotherapy that with our present state of knowledge appears to have considerable potential. For this potential to be realised there are problems that need to be addressed and resolved.

(i) *Antigenic heterogeneity* The erratic distribution of binding sites throughout a tumour is likely to produce a similarly erratic deposition of energy in the tumour cell population. There are a number of approaches being adopted to try to achieve a more uniform radiation dose. In the forseeable future it would appear that success is most likely to come from the use of a combination of McAbs directed against different epitopes or from the production of new McAbs to less heterogeneously distributed tumour-associated antigens.

(ii) *Specificity of antibody* Many of the presently available antibodies localize in areas other than the tumour. It is important that the search continues for carriers that will preferentially localize in tumours.

(iii) *Fractionation of radiation* With external irradiation fractionation of the dose is used to give the normal tissues time to repair. The same principle will apply with $\beta$ emitters – but not with $\alpha$ emitters, and probably not with Auger cascades. The difficulty with fractionation of the dose with RIT is that at present we have the major problem of the antigenicity of the carrier. The pragmatic and seemingly successful approach to this problem made by Order and his colleagues (1985) has been to use a succession of polyclonal antibodies raised in different species of animal. An obvious alternative is to employ genetic engineering to build carrier molecules with a minimum of antigenicity – although we are still left with the antigenicity of the idiotype. In the more distant future we may be able to approach the problem by blocking either the patients' recognition of the foreign antibody or the ability to respond to it.

(iv) *Access of antibody to target antigen* An estimate can be made of the radioactivity in a tumour mass using $\gamma$ scintigraphy or positron-emission tomography. This does not however provide information on the microscope distribution of activity, which is essential before any estimate can be made of the proportion of tumour cells likely to be sterilized or give advice on the most appropriate radionuclide. This information can only be obtained from biopsies of the tumour in the days following the injection of labelled antibody – and such material is rarely available. This represents a major gap in our knowledge. We can make the assumption that the capillaries in a rapidly growing tumour will be relatively permeable to antibody – but such may not be the case in the common slow growing tumours, and we may need to seek ways for improving tumour capillary permeability. Efforts are continually being made to reduce the size of the carrier molecule in an attempt to improve access to extravascular sites. The molecule shape and charge need also to be considered.

As with all new forms of tumour therapy the initial optimism will become tempered with experience. Our aim must therefore be to try to identify as quickly as possible those categories of patients who will benefit by the inclusion of RIT in their treatment regimen.

We particularly wish to acknowledge the valuable assistance provided by numerous discussions with Mrs A. Harrison of this Unit. Dr J. Savage suggested the term zetotherapy.

**References**

ALBINO, A.P., LLOYD, O.K., HOUGHTON, A.N., OETTGEN, H.F. & OLD, L.J. (1981). Heterogeneity in surface antigen and glycoprotein expression of cell lines derived from different melanoma metastases of the same patient. *J. Exp. Med.*, **154,** 1764.

BADGER, C.C., KROHN, K.A., PETERSON, A.V., SCHULMAN, H. & BERNSTEIN, I.D. (1985). Experimental radiotherapy in murine lymphoma with $^{131}$I-labelled anti-Thy 1.1 monoclonal antibody. *Cancer Res.*, **45,** 1536.

BATEMAN, W.J., VAUGHAN, A.T.M. & BROWN, G. (1983). Selective alpha-irradiation of experimental tumours using astatine-211 labelled monoclonal antibodies. *Proc. 7th Int. Cong. Radiat. Res.*, Amsterdam D1-03. (*Abstract*).

BEGENT, R.H.J., KEEP, P.A., GREEN, A.J. & 6 others (1982). Liposomally entrapped second antibody improves tumour imaging with radiolabelled (first) antitumour antibody. *Lancet*, **ii,** 739.

BEGENT, R.H.J., GREEN, A.J., CHESTER, K.A., KEEP, P.A., SEARLE, F. & BAGSHAWE, K.D. (1984). The use of second antibody to improve selective localisation: Clinical studies. *Br. J. Cancer*, **50,** 556. (*Abstract*).

BLÖCHER, D. (1981). Inaugural-dissertation. Strahleninduzierte DNA-Doppelstrangbrüche in Ehrlich Ascites Tumorzellen und ihre mögliche Bedeutung für das Zellüberleben. *Ph.D. Thesis Frankfurt University*. Table 6.3, p. 123.

BLÖCHER, D. (1982). DNA double strand breaks in Ehrlich ascites tumour cells at low doses of X-rays. I. Determination of induced breaks by centrifugation at reduced speeds. *Int. J. Radiat. Biol.*, **42,** 317.

BURAGGI, G.L., CALLEGARO, L., MARIANI, G. & 12 others (1985). Imaging with $^{131}$I-labelled monoclonal antibodies to a high-molecular-weight melanoma-associated antigen in patients with melanoma: Efficacy of whole immunoglobulin and its $F(ab')_2$ fragments. *Cancer Res.*, **45,** 3378.

BURCHIEL, S.W., MARTIN, J.C., IMAI, K., FERRONE, S. & WARNER, N.L. (1982). Heterogeneity of HLA-A, B, Ia-like, and melanoma-associated antigen expression by human melanoma cell lines analysed with monoclonal antibodies and flow cytometry. *Cancer Res.*, **42,** 4110.

BURKI, H.J., ROOTS, R., FEINENDEGEN, L.E. & BOND, V.P. (1973). Inactivation of mammalian cells after disintegrations of $^{3}$H or $^{125}$I in cell DNA at $-196$°C. *Int. J. Radiat. Biol.*, **24,** 363.

CARRASQUILLO, J.A., KROHN, K.A., BEAUMIER, P. & 5 others (1984). Diagnosis of and therapy for solid tumours with radio-labeled antibodies and immune fragments. *Cancer Treat. Rep.*, **68,** 317.

COBBOLD, S.P. & WALDMANN, H. (1984). Therapeutic potential of monovalent monoclonal antibodies. *Nature*, **308,** 460.

COURTENAY-LUCK, N. & 19 others (1984). Antibody-guided irradiation of malignant lesions: Three cases illustrating a new method of treatment. *Lancet*, **i,** 1441.

DeNARDO, S.J., ERICKSON, K.L., BENJAMAN, E. & 3 others (1982). Monoclonal antibodies for radiation therapy of melanoma. In *Nuclear Medicine and Biology*, Raynaud, (ed) p. 182. Pergamon Press: Paris.

DeNARDO, S.J., DeNARDO, G.L., PENG, J-S. & COLCHER, D. (1983). Monoclonal antibody radiopharmaceuticals for cancer radioimmunotherapy. In *Radioimmuno-imaging and Radioimmunotherapy*, Burchiel & Rhodes, (eds) p. 405. Elsevier: New York.

DIENER, E., DINER, U.E., SINHA, A., XIE, S. & VERGIDIS, R. (1986). Specific immunosuppression by immuno-toxins containing daunomycin. *Science*, **231,** 148.

DILLMAN, R.O., BEAUREGARD, J.C., SOBOL, R.E. & 4 others (1984). Lack of radioimmunodetection and complications associated with monoclonal anticarcino-embryonic antigen antibody cross-reactivity with an antigen on circulating cells. *Cancer Res.*, **44,** 2213.

EDWARDS, P.A.W. (1985). Heterogenous expression of cell-surface antigens in normal epithelia and their tumours, revealed by monoclonal antibodies. *Br. J. Cancer*, **51**, 149.

EDWARDS, P.A.W., SKILTON, R.A., PAYNE, A.W.R. & ORMEROD, M.G. (1985). Antigenic heterogeneity of breast cell lines detected by monoclonal antibodies and its relationship with the cell cycle. *J. Cell Sci.*, **73**, 321.

EPENETOS, A.A., COURTENAY-LUCK, N., PICKERING, D. & 4 others (1985). Antibody irradiation of brain glioma by arterial infusion of radioactive monoclonal antibody against epidermal growth factor receptor and blood group A antigen. *Br. Med. J.*, **290**, 1463.

ETTINGER, D.S., DRAGON, L.H., KLEIN, J., SGAGIAS, M. & ORDER, S.E. (1979). Isotopic immunoglobulin in an integrated multimodal treatment program for a primary liver cancer: A case report. *Cancer Treat. Rep.*, **63**, 131.

ETTINGER, D.S., ORDER, S.E., WHARAM, M.D. & 3 others (1982). Phase I-II study of isotopic immunoglobulin therapy for primary liver cancer. *Cancer Treat. Rep.*, **66**, 289.

FARGION, S., CARNEY, D., MULSHINE, J. & 6 others (1986). Heterogeneity of cell surface antigen expression of human small cell lung cancer detected by monoclonal antibodies. *Cancer Res.*, **46**, 2633.

FAWWAZ, R.A., WANG, T.S.T., SRIVASTAVA, S.C. & 4 others (1984). Potential palladium-109-labeled anti-melanoma monoclonal antibody for tumor therapy. *J. Nucl. Med.*, **25**, 796.

FEINENDEGEN, L.E. (1975). Biological damage from the Auger effect: Possible benefits. *Radiat. Environ. Biophys.*, **12**, 85.

FOSTER, C.S., EDWARDS, P.A.W., DINSDALE, E.A. & NEVILLE, A.M. (1982*a*). Monoclonal antibodies to the human mammary gland. II. Distribution of determinants in breast carcinomas. *Virchows Arch.* (*Pathol. Anat.*), **394**, 295.

FOSTER, C.S., EDWARDS, P.A.W., DINSDALE, E.A. & NEVILLE, A.M. (1982*b*). Monoclonal antibodies to the human mammary gland. I. Distribution of determinants in non-neoplastic mammary and extra mammary tissues. *Virchows Arch.* (*Pathol. Anat.*), **394**, 279.

FRANK, M.M., LAWLEY, T.J., HAMBURGER, M.I. & BROWN, E.J. (1983). Immunoglobulin Fc receptor-clearance in autoimmune diseases. *Ann. Int. Med.*, **98**, 206.

GLENNIE, M.J. & STEVENSON, G.T. (1982). Univalent antibodies kill tumour cells *in vitro* and *in vivo*. *Nature*, **295**, 712.

GOLDENBERG, D.M., GAFFAR, S.A., BENNETT, S.J. & BEACH, J.L. (1981). Experimental radioimmunotherapy of a xenografted human colonic tumour (GW-39) producing CEA. *Cancer Res.*, **41**, 4354.

GREINER, J.W., HAND, P.H., NOGUCHI, P. & 3 others (1984). Enhanced expression of surface tumor-associated antigens in human breast and colon tumor cells after recombinant human leukocyte α-interferon treatment. *Cancer Res.*, **44**, 3208.

HAGAN, P.L., HALPERN, S.E. & CHEN, A. (1983). Comparison of In-111 labelled Fab and whole In-111 anti-CEA monoclonal antibody (MoAb) in normal mice – human colon tumor models. *J. Nucl. Med.*, **24**, P77.

HAMBLIN, T.J., ABDUL-AHAD, A.K., GORDON, J., STEVENSON, F.K. & STEVENSON, G.T. (1980). Preliminary experience in treating lymphocytic leukemia with antibody to immunoglobulin idiotypes on the cell surfaces. *Br. J. Cancer*, **42**, 495.

HAND, P.H., NUTI, M., COLCHER, D. & SCHLOM, J. (1983). Definition of antigenic heterogeneity and modulation among human mammary carcinoma cell populations using monoclonal antibodies to tumor-associated antigens. *Cancer Res.*, **43**, 728.

HARRISON, A. & ROYLE, L. (1984). Preparation of a $^{211}$At-IgG conjugate which is stable *in vivo*. *Int. J. Appl. Radiat. Isot.*, **35**, 1005.

HARRISON, A. & ROYLE, L. (1986). The efficacy of a $^{211}$At-monoclonal antibody in the treatment of a murine T cell lymphoma. *Natl Cancer Inst. Mongr.* (In press).

HIRANO, A. & ZIMMERMAN, H.M. (1972). Fenestrated blood vessels in a metastatic renal carcinoma in the brain. *Lab. Invest.*, **26**, 465.

HNATOWICH, D.J., VIRZI, F. & DOHERTY, P.W. (1985). DTPA-coupled antibodies labeled with yttrium-90. *J. Nucl. Med.*, **26**, 503.

HOFER, K.G. (1980). Radiation biology and potential therapeutic applications of radionuclides. *Bull. Cancer (Paris)*, **67**, 343.

KLEIN, J.L., LING, M.N., LEICHNER, P.K. & 3 others (1985). A model system that predicts effective half-life for radiolabeled antibody therapy. *Int. J. Radiat. Oncol. Biol. Phys.*, **11**, 1489.

KOZAK, R.W., ATCHER, R.W., GANSOW, O.A. & 3 others (1986). Bismuth-212-labelled anti-Tac monoclonal antibody: α-particle-emitting radionuclides as modalities for radioimmunotherapy. *Proc. Natl Acad. Sci.*, **83**, 474.

KUFE, D.W., NADLER, L., SARGENT, L. & 5 others (1983). Biological behavior of human breast carcinoma-associated antigens expressed by cellular proliferation. *Cancer Res.*, **43**, 851.

LARSON, S.M., CARRASQUILLO, J.A., KROHN, K.A. & 8 others (1983). Localisation of $^{131}$I-labelled p97-specific Fab fragments in human melanoma as a basis for radiotherapy. *J. Clin. Invest.*, **72**, 2101.

LEICHNER, P.K., KLEIN, J.L., SIEGELMAN, S.S., ETTINGER, D.S. & ORDER, S.E. (1983). Dosimetry of $^{131}$I-labelled antiferritin in hepatoma: Specific activities in the tumor and liver. *Cancer Treat. Rep.*, **67**, 647.

LEICHNER, P.K., KLEIN, J.L., FISHMAN, E.K., SELIGMAN, S.S., ETTINGER, D.S. & ORDER, S.E. (1984). Comparative tumour dose from $^{131}$I-labelled polyclonal anti-ferritin, anti-AFP, anti-CEA in primary liver cancers. *Cancer Drug Delivery*, **1**, 321.

LIBER, H.L., LEMOTTE, P.K. & LITTLE, J.B. (1983). Toxicity and mutagenicity of X-rays and $^{125}$IdUrd or $^{3}$HTdR incorporated in the DNA of human lymphoblast cells. *Mutat. Res.*, **111**, 387.

LUDATSCHER, R.M., GELLEI, B. & BARZILAI, D. (1979). Ultrastructural observations on the capillaries of human thyroid tumours. *J. Pathol.*, **127**, 57.

MARTIN, R.F. (1977). Induction of double-stranded breaks in DNA by binding with an $^{125}$I-labelled acridine. *Int. J. Radiat. Biol.*, **32**, 491.

MARTIN, R.F., BRADLEY, T.R. & HODGSON, G.S. (1979). Cytotoxicity of an $^{125}$I-labelled DNA-binding compound that induces double-strand DNA breaks. *Cancer Res.*, **39**, 3244.

MEEKER, T.C., LOWDER, J., MALONEY, D.G. & 4 others (1985). A clinical trial of anti-idiotype therapy for B cell malignancy. *Blood*, **65**, 1349.

NADLER, L.M., STASHENKO, P., HARDY, R. & 5 others (1980). Serotherapy of a patient with a monoclonal antibody directed against a human lymphoma-associated antigen. *Cancer Res.*, **40**, 3147.

NATALI, P.G., CAVALIERE, R., BIGOTTI, A. & 5 others (1983). Antigenic heterogeneity of surgically removed primary and autologous metastatic human melanoma lesions. *J. Immunol.*, **130**, 1462.

ORDER, S.E., KLEIN, J.L., ETTINGER, D. & 3 others (1980*a*). Use of isotopic immunoglobulin in therapy. *Cancer Res.*, **40**, 3001.

ORDER, S.E., KLEIN, J.L., ETTINGER, D. & 4 others (1980*b*). Phase I-II study of radiolabelled antibody integrated in the treatment of primary hepatic malignancies. *Int. J. Radiat. Oncol. Biol. Phys.*, **6**, 703.

ORDER, S.E., KLEIN, J.L. & LEICHNER, P.K. (1981). Antiferritin IgG antibody for isotopic cancer therapy. *Oncol.*, **38**, 154.

ORDER, S.E., STILLWAGON, S.B., KLEIN, J.L. & 10 others (1985). Iodine 131 antiferritin, a new treatment modality in hepatoma: A Radiation Therapy Oncology Group study. *J. Clin. Oncol.*, **3**, 1573.

PECTASIDES, D., STEWART, S., COURTENEY-LUCK, & 10 others (1986). Antibody-guided irradiation of malignant pleural and pericardial effusions. *Br. J. Cancer*, **53**, 727.

PRIMUS, F.J., BENNETT, S.J., KIM, E.E. & 3 others (1980). Circulating immune complexes in cancer patients receiving goat radiolocalizing antibodies to carcinoembryonic antigen. *Cancer Res.*, **40**, 497.

ROSTOCK, R.A., KLEIN, J.L., LEICHNER, P.K. & ORDER, S.E. (1984). Distribution of and physiological factors that affect $^{131}$I-antiferritin tumour localization in experimental hepatoma. *Int. J. Radiat. Oncol. Biol. Phys.*, **10**, 1135.

SAHAGAN, B.G., DORAI, H., SALTZGABER-MULLER, J. & 9 others (1986). Genetically engineered murine/human chimeric antibody retains specificity for human tumor-associated antigen. *J. Immunol.* (In press).

SIKORA, K., ALDERSON, T., NETHERSELL, A. & SMEDLEY, H. (1985). Tumour localisation by human monoclonal antibodies. *Med. Oncol. Tumor Pharmacother.*, **2**, 77.

SUTER, L., BRÜGGEN, J., BRÖCKER, E.B. & SORG, C. (1985). A tumor-associated antigen expressed in melanoma cells with low malignant potential. *Int. J. Cancer*, **35**, 787.

VAUGHAN, A.T.M., BATEMAN, W.J. & FISHER, D.R. (1982). The *in vivo* fate of a $^{211}$At labelled monoclonal antibody with known specificity in a murine system. *Int. J. Radiat. Oncol. Biol. Phys.*, **8**, 1943.

WARENIUS, H.M., GALFRE, G., BLEEHEN, N.M. & MILSTEIN, C. (1981). Attempted targeting of a monoclonal antibody in a human tumour xenograft system. *Eur. J. Cancer Clin. Oncol.*, **17**, 1009.

WRIGHT, G.L., BECKETT, M.L., STARLING, J.J. & 4 others (1983). Immunohistochemical localisation of prostate carcinoma-associated antigens. *Cancer Res.*, **43**, 5509.

ZALCBERG, J.R., THOMPSON, C.H., LICHTENSTEIN, M. & McKENZIE, I.F.C. (1984). Tumor immunotherapy in the mouse with the use of $^{131}$I-labelled monoclonal antibodies. *J. Natl Cancer Inst.*, **72**, 697.